# Mutated tumor alleles are expressed according to their DNA frequency

**DOI:** 10.1038/srep04743

**Published:** 2014-04-22

**Authors:** John C. Castle, Martin Loewer, Sebastian Boegel, Arbel D. Tadmor, Valesca Boisguerin, Jos de Graaf, Claudia Paret, Mustafa Diken, Sebastian Kreiter, Özlem Türeci, Ugur Sahin

**Affiliations:** 1 TRON gGmbH - Translational Oncology at Johannes Gutenberg-University Medical Center gGmbH, Langenbeckstr. 1, Building 708, 55131 Mainz, Germany; 2 University Medical Center of the Johannes Gutenberg-University Mainz, 55131, Mainz, Germany; 3 BioNTech AG, Kupferbergterrasse 17-19, 55131 Mainz, Germany

## Abstract

The transcription of tumor mutations from DNA into RNA has implications for biology, epigenetics and clinical practice. It is not clear if mutations are in general transcribed and, if so, at what proportion to the wild-type allele. Here, we examined the correlation between DNA mutation allele frequency and RNA mutation allele frequency. We sequenced the exome and transcriptome of tumor cell lines with large copy number variations, identified heterozygous single nucleotide mutations and absolute DNA copy number, and determined the corresponding DNA and RNA mutation allele fraction. We found that 99% of the DNA mutations in expressed genes are expressed as RNA. Moreover, we found a high correlation between the DNA and RNA mutation allele frequency. Exceptions are mutations that cause premature termination codons and therefore activate nonsense-mediated decay. Beyond this, we did not find evidence of any wide-scale mechanism, such as allele-specific epigenetic silencing, preferentially promoting mutated or wild-type alleles. In conclusion, our data strongly suggest that genes are equally transcribed from all alleles, mutated and wild-type, and thus transcribed in proportion to their DNA allele frequency.

## Background

Cancer is caused by DNA mutations resulting in single nucleotide variations (SNVs), structural rearrangements and copy number variations (CNVs)^1^,^2^. A SNV frequently occurs on a single allele; the impact of a heterozygous SNV will depend on whether the SNV-containing allele is transcribed to RNA. Indeed, a non-transcribed SNV, non-synonymous or silent, could be phenotypically invisible. Clinical therapy-selection biomarkers often assay mutations using DNA as an analyte, such as KRAS assays designed to identify responders to anti-EGFR monoclonal antibody therapy^3^. However, if the wild-type allele is selectively transcribed, the mutation may not have therapeutic impact and the merit of using a DNA-based assay for clinical decision-making may be problematic.

Given a heterozygous non-synonymous SNV, the phenotypical impact will depend on whether the SNV-containing allele is transcribed to RNA. A heterozygous SNV in a polyploidy region could be functionally invisible due to multiple mechanisms. The mutation-containing allele could be effectively silenced by epigenetic and chromatin modifying mechanisms. Chromosome X in females, for example, contains silenced and non-silenced alleles. Further, a SNV-containing transcript could activate RNA surveillance mechanisms and cause rapid degradation of the mutation-containing transcript. Nonsense-mediated decay (NMD) surveillance, for example, scans transcripts for the presence of a premature termination codons (PTCs) before the last exon and, when found, initiates degradation of such transcripts^4^. NMD surveillance has been extensively studied in RNA splicing^5^, where it removes aberrantly spliced transcripts, and in microsatellite instability colorectal cells, where NMD blockage up-regulates genes containing somatic mononucleotide repeat mutations causing frameshift open reading frames^6^. If and to which extent this happens has not been systematically analyzed.

High throughput profiling technologies, such as next generation sequencing (NGS), have enabled the determination of DNA and RNA mutation allele frequency and copy number. Increased DNA copy number results, in general, in increased gene expression^7^. The allowable DNA allele frequencies of a mutation are discrete values based on the underlying genomic copy number. In haploid regions, mutations have 100% allele frequency. In diploid regions, mutations have allele frequencies of 50 or 100%, in triploid regions it is 33, 66, or 100%, and in tetraploid regions 25, 50, 75 or 100%. Experimentally, genomic copy number can be effectively determined from SNP-monitoring oligonucleotide microarrays and NGS genome and exome profiling^8^,^9^. Integration of the allele fraction of heterozygous germline SNPs both enables identification of regions containing imbalanced alleles, including loss of heterozygosity (LOH) and mutant allele-specific DNA amplification^10^, and determination of tumor cell purity and absolute tumor ploidy^11^,^12^. Integration of the DNA mutation allele frequency further improves mutation detection and delineation of tumor evolution^13^,^14^,^15^,^16^.

Studies have examined imbalanced allele RNA expression^17^. Imprinted alleles can be identified with NGS RNA-Seq reads^18^. In tumors, heterozygous SNPs can be transcribed at different abundances than in normal cells^19^, and the genes with imbalanced SNP expression are associated with higher DNA copy number. This raises the possibility that allele-imbalanced DNA amplification leads to a corresponding imbalance in the RNA levels of SNPs. Indeed, a study examining the DNA and RNA mutation allele frequencies in four genes found high correlation between DNA and RNA frequencies^13^.

Surprisingly, given the importance to biology and clinical oncology, a genome-wide study of the relationship between DNA and RNA mutation allele frequency in tumor samples has not been done. The question of whether mutated alleles are generally transcribed and, if so, how they are represented at the transcript level – imbalanced, compensated to a diploid setting or proportional to the DNA dosage – has not been systematically analyzed with genome-wide approaches.

To answer these questions, we analyzed the presence of mutations in DNA and their corresponding RNA expression on an allele-specific level with a genome-wide approach. We sequenced the broadly used B16F10, 4T1 and CT26 mouse tumor cell lines. These cell lines have the advantages that they contain large copy number variations, and thus varying levels of DNA mutation allele frequency, and are homogeneous cells without normal cell contamination. We used NGS to profile DNA and RNA and developed data processing algorithms to identify mutations, define absolute copy number and determine both DNA and RNA mutation allele frequencies. Our findings provide the first systematic genome-wide study of DNA and RNA mutation allele frequency in tumor samples and therewith demonstrate that mutations are both transcribed and they are transcribed in proportion to their DNA allele frequency.

## Results and discussion

Using the replicate cancer and germline exome resequencing from three different mouse tumor cell lines, we identified high confidence single nucleotide point mutations (SNVs) and DNA copy number variations (CNVs). There are 3023 SNVs in mouse colorectal carcinoma CT26 cells compared to BALB/cJ, 908 in mouse melanoma B16F10 cells compared to C57BL/6, and 293 in mouse breast tumor 4T1 cells compared to BALB/cJ. These results agree with reports demonstrating that human melanomas and colorectal tumors have among the highest mutation rates of primary tumors while breast tumors average an order of magnitude fewer mutations^26^.

**Figure 1:**
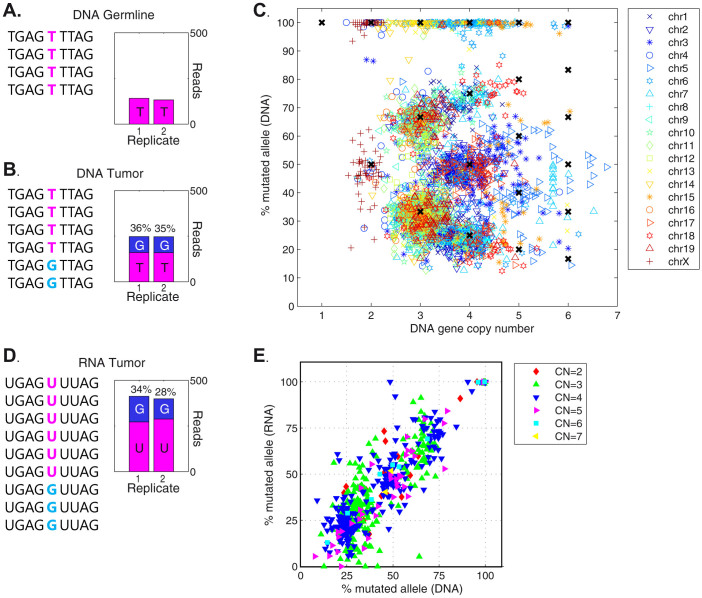
DNA and RNA mutation allele frequency and copy number in the CT26 mouse colon cancer cell line. (A and B): the sequence content and normalized read counts from the (A) BALB/cJ germline and (B) CT26 tumor DNA reads at the exemplary T > G Eif4g2 mutation locus (chr7, 118,222,833). Four nucleotides are shown on either side of the mutation. The exome of each sample (germline and tumor) was sequenced in duplicate. The normalized total number of reads overlapping the mutation locus is shown for each sample and replicate. The replicates show similar total counts and mutation frequencies. (C) The DNA gene copy number and DNA mutation allele frequency of the 3023 SNVs in CT26. Symbols and colors represent different chromosomes. Black crosses mark allowable mutation allele frequency and copy number combinations. The mutations with 100% allele frequency are homozygous mutations. (D) The sequence content and normalized read counts from the CT26 tumor RNA reads at the T > G Eif4g2 mutated locus. The CT26 transcriptome was sequenced in duplicate; the replicates show similar total counts and mutation frequencies. (E) The DNA and RNA mutation allele frequency for the 697 SNVs covered by at least 10 reads. SNVs are colored according to identified gene DNA copy number (CN), as determined in (C).

We calculated the DNA mutation allele frequency using the sequence content of the reads overlapping each SNV. As an example, the gene Eif4g2 (eukaryotic translation initiation factor 4 gamma 2) is highly expressed in CT26 cells and contains a T > G heterozygous somatic mutation. The normalized read counts associated with the Eif4g2 mutation for the two replicates are 141 and 133 in the germline sample and 248 and 247 in the CT26 sample (Figure 1A and B). The G mutation is not found in the germline reads (Figure 1A) whereas it is found in 35% and 36% of the DNA reads from the tumor (Figure 1B).

Using the read counts and frequencies for all genes and mutations, respectively, we simultaneously determined the allele frequency of each mutation, the absolute DNA copy number of each gene and the mean ploidy for the sample. We found that the CT26 genome has many triploid, tetraploid and pentaploid regions and many regions with homozygous mutations, suggesting multiple LOH events from the onco-transformation or inbreeding (Figure 1C)^27^. Further, we observed that mutation allele frequencies correctly occur at the distinct frequencies allowed by the DNA copy number (Figure 1C). For example, the mutations in the copy number 4 regions occur at 25, 50, 75 or 100%. With the computational platform working, we determined the DNA mutation allele frequency for each SNV. We found, for example, that the Eif4g2 locus falls in a region with copy number 3. Based on the observed 35% and 36% mutation allele frequencies, the G mutation occurs on one allele and the wild-type T occurs on two alleles.

We determined the RNA mutation allele frequency using the NGS RNA-Seq reads overlapping the identified SNVs. We selected SNVs for which there were at least 10 overlapping RNA reads, which comprised 697 SNVs in CT26. The determination of RNA mutation allele frequencies is sensitive to the read alignment algorithm^28^,^29^. We compared multiple methods and selected the STAR algorithm due to its ability to effectively align reads containing mismatches^21^. Examining the reads overlapping the exemplar Eif4g2 T > G mutation, 34% and 28% contain the mutation (Figure 1D).

Genome-wide analysis using this procedure revealed that of the 697 mutations in CT26 expressed genes, 688 mutations are present in the RNA reads and only 9 are absent. Second, all homozygous DNA mutations are also correctly homozygous at the RNA level. Third, the DNA and RNA mutation allele frequencies correlate remarkably well (Figure 1E, r^2^ = 0.82). These results show that a) CT26 mutations in expressed genes are transcribed with over 99% likelihood and b) the mutations are transcribed in equal proportion to the underlying DNA mutation allele frequency.

**Figure 2:**
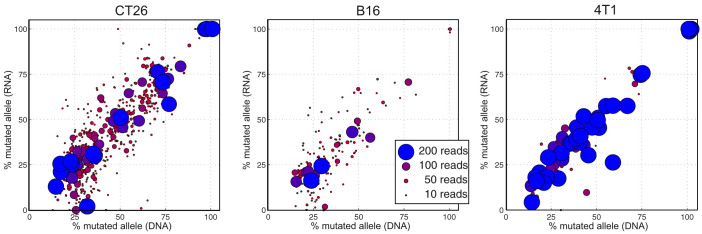
The DNA and RNA mutation allele frequency in CT26, B16F10 and 4T1 cells. The marker color and size are determined by the total number of RNA reads (mutation containing plus wild-type containing reads) that overlap the SNV coordinate.

**Figure 3:**
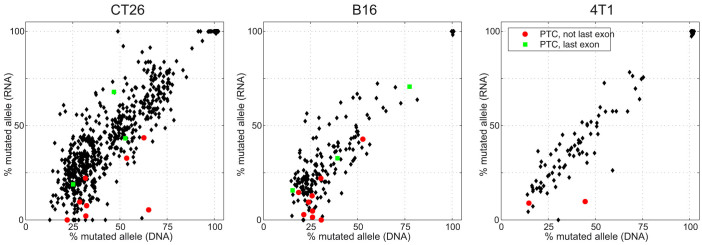
The impact of PTC-containing mutations on the CT26, B16F10 and 4T1 transcriptomes. Red circles mark PTC-causing mutations in non-last-exons; green squares represent PTC-causing mutations in last exons.

We repeated this analysis for the B16F10 and 4T1 samples (Figures 2 and 3). There were 182 mutations with at least 10 RNA reads in B16F10. 179 of the 182 mutations are expressed (98%) and the RNA and DNA mutation allele frequencies correlate well (r^2^ = 0.75). In 4T1, there were 101 transcribed mutation loci with at least 10 RNA reads. 100 of the 101 mutations are expressed (99%) and the RNA and DNA mutation allele frequencies correlate very well (r^2^ = 0.94). Together, 967 of 980 mutations in expressed genes are expressed (99%) and the RNA and DNA mutation allele frequencies correlate highly.

**Figure 4:**
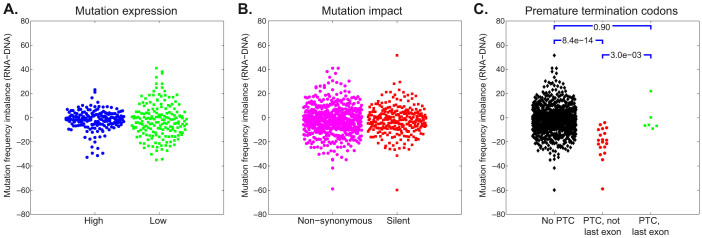
Mutation frequency imbalance (RNA-DNA). (A) A comparison of high and low expressed mutations based on mutations with at least 65 read coverage (blue, n = 179) or less than 15 read coverage (green, n = 180). The cutoffs were selected such that the sets have similar number of members. The means are similar but the variance in the low expression group is much higher. (B) The mutation frequency imbalance of non-synonymous (magenta, n = 596) and silent (red, n = 286) mutations. (C) The non-PTC (black, n = 955), PTC not-last-exon (red, n = 19) and PTC last-exon (green, n = 6) mutation sets and p-values.

Next, we examined the outliers for evidence of biological processes. We developed a metric for measuring RNA versus DNA mutation allele imbalance: 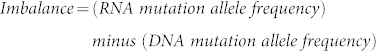


For most mutations, the RNA and DNA mutation allele fractions are similar and the imbalance is near zero. Highly expressed mutation with more reads, correlate more strongly and have a smaller imbalance variance, as can be seen when comparing the large and small points in Figure 2. Indeed, the mean r^2^ values are 0.88 and 0.78 and the standard deviations are 8.2 and 14.0 for the subsets of mutations with more than 65 or less than 15 reads coverage, respectively (Figure 4A). This is likely because the RNA allele frequency is more accurate with higher coverage^30^. 39 of 980 mutations (4%) have an absolute imbalance score greater than 25 and all 39 have relative low expression. We compared the mutation allele frequency imbalance in silent and non-synonymous mutations and found no difference (p = 0.86) (Figure 4B). We expected heterozygous mutations on the X chromosome, having 50% DNA allele frequencies, to have either 0% or 100% RNA allele frequencies due to inactivated X alleles. However, the RNA frequencies of heterozygous X chromosome mutations were centered near 50%. This is likely because 14 of the 15 identified heterozygous X chromosome SNVs were found in the CT26 cells and CT26 cells transcribe from both alleles, having lost the inactivated X allele^27^. Imprinting causes selective allele transcription^18^. Across the three cell lines, we found only two SNVs in known imprinted genes^31^, in genes Atp10a and Plagl1, and neither SNV allele frequency is imbalanced. This suggests that these genes are not imprinted in these tumor samples or that the mutations occurred on amplified, non-imprinted alleles.

We found that SNVs causing PTCs in non-last exons were expressed at significantly lower frequencies than predicted from the DNA allele frequencies (Figures 3 and 4C). SNVs causing PTCs in last exons, however, were expressed at the DNA-predicted allele frequencies. The p-value between the “no PTC” (black) and “PTC, not last exon” (red) imbalances is 8e-14, whereas the difference between the “no PTC” and “PTC in last exon” (green) is insignificant (p = 0.9). Both observations are in agreement with the established mechanism of NMD surveillance, in which NMD scans transcripts for PTCs occurring before the last exon and initiates degradation of such transcripts.

## Conclusions

Cancer cells contain DNA mutations and the RNA expression of heterozygous non-synonymous mutations has impacts on biological, epigenetic and medical questions. While several studies have examined DNA allele frequency, there has not been a genome-wide examination of the translation of DNA mutations. Thus, the objective of this study was to determine if mutations are, in general, transcribed and, if so, whether they are selectively transcribed.

Unlike somatic mutation detection which requires an inter-sample comparison (tumor versus normal), the determination of mutation allele frequencies is a self-normalizing intra-sample comparison and is thus relatively robust to sample handling and laboratory workflows. However, as the mutation-containing reads contain a mismatch to the reference sequence, they are more difficult to computationally align than wild-type reads. Indeed, we found that different alignment algorithms introduced significant systematic biases in the determination of allele frequencies. We preferred the STAR algorithm for RNA read alignment.

One of our key findings is that 99% of the point mutations in expressed genes are transcribed. Thus, the likelihood that a mutation in a transcribed gene will be transcribed to RNA is high. Second, we found that mutations are transcribed in equal proportion to their DNA allele frequency (up to r^2^ = 0.94) and thus, RNA and DNA dosages are matched. Third, we identified nonsense-mediated decay of mutations resulting in premature stop codons prior to the last exon as a primary mechanism to introduce imbalances of mutated versus wildtype alleles. Along this line, we did not observe an influence of X-inactivation or imprinting, although this was likely due to the small number of mutations in imprinted genes. We did not find a difference between silent and non-synonymous mutations. Nor did we find surveillance, epigenetic or other compensatory feedback mechanism that selectively transcribes or silences mutation alleles. As investigation of mutations in regulatory regions outside of transcripts, such as in transcription factor binding sites (TFBSs), requires genome instead of exome resequencing, the impact of the potential cis-acting mutations on allele-specific transcription remains unclear.

The results here are consistent with transcription occurring equally from all alleles, mutated and wild-type. Outside of NMD, we did not find evidence for a general surveillance or epigenetic silencing mechanism that acts to degrade or prevent transcription of entire classes of mutation-containing transcripts, suggesting that the default tumor state is to equally transcribe from mutated and wild-type alleles. For cancer patients, this provides support for DNA-based mutation-detection assays for patient stratification, which will gain increasing relevance with mutation-targeting immunotherapies showing pre-clinical proof-of-concept^32^ and entering clinical trials, such as trial NCT02035956.

## Methods

*Samples:* C57BL/6 and BALB/cJ mice (Charles River) were kept in accordance with legal and ethical policies on animal research. All animal protocols were approved by the government of Rheinland-Palatinate's Animal Care Committee, Koblenz, Germany. Germline BALB/cJ and C57BL/6 DNA samples were extracted from mouse tail. B16F10 melanoma and CT26 colon carcinoma cells were purchased from ATCC (Manassas, USA); 4T1 breast tumor cells were purchased from Caliper Life Sciences (4T1-luc2-tdTomato, product 125669), who derived them from ATCC CRL-2539. B16F10 was originally derived from a C57BL/6 mouse. CT26 was derived from BALB/cJ mouse, and 4T1 from a BALB/cfC3H mouse.

*NGS sequencing and data processing*: replicate exome capture for DNA resequencing was performed using the Agilent Sure-Select mouse whole-exome solution-based capture assay. Oligo (dT) RNA was isolated from each tumor cell line (B16F10, CT26 and 4T1) in replicate and was prepared for gene expression profiling. Libraries were sequenced on an Illumina HiSeq2000. DNA reads were aligned with bwa^20^ (version 0.5.8c, default options). Ambiguous reads mapping to multiple locations of the genome were removed. RNA reads aligned with STAR^21^ (version 2.1.4a, default options). Individual exome replicates contained an average of 113 million reads per sample and individual RNA-Seq replicates contained an average of 26 million reads per sample.

*Mutation identification:* single nucleotide mutations (SNVs) that were identified by all algorithms samtools^22^, Mutect^23^ and SomaticSniper^24^ (all with default options) and found in both replicates were further filtered using binomial filters that eliminated erroneous tumor-only coverage artifacts and thus decreased the likelihood that a mutation was classified as somatic due to lack of coverage in the germline sample.

*DNA copy number:* absolute allele copy number, tumor purity and mutation allele fraction were simultaneously determined using a novel algorithm that assumes a) that mutation allele fraction can take only discrete values in tumor cells based on allele copy number and b) that the relative tumor to germline number of exome-seq reads mapping to a gene locus is proportional to locus copy number^25^.

*Mutation source*: the identified SNVs represent variations between a tumor genome in cells derived from an historic mouse and a modern inbred mouse of the same strain. For example, the CT26 cells were derived from a BALB/c mouse in 1975 and the normal cells sequenced here were from a BALB/cJ mouse in 2011. As such, the SNVs include both somatic mutations associated with the onco-transformation and genetic drift in the inbred mice.

*DNA and RNA mutation allele fraction* were determined by examining the nucleotide sequence of the reads overlapping each SNVs. The mutation allele fraction was calculated as the number of mutation-containing reads divided by all reads overlapping the SNV. Mutations were considered if at least ten DNA and ten RNA reads overlapped the genomic position. A minimum cutoff of ten was selected to increase the accuracy when determining mutation allele percentages: a higher cutoff would lead to higher accuracy but would decrease the total number of data points. The results obtained when using higher or lower cutoffs are very similar. Frequencies from replicates were combined using error weighted averaging based on uncertainties from the binomial distribution and the number of reads for the frequency measurement. Essentially, frequencies from samples with more reads were given more weight.

*Statistics* were calculated using Matlab software packages. Correlation coefficients represent Pearson's linear correlation coefficient. P-values were calculated with a two-sample T-test testing whether the hypothesis that the two sample sets come from the same normal distribution be rejected at the 5% significance level.

An additional table contains annotated CT26, B16 and 4T1 mutation lists. NGS fastq files for B16, CT26, 4T1 (4T1-luc2-tdTomato), C57BL/6 and BALB/cJ are available from the European Nucleotide Archive (ENA) as PRJEB5797, PRJEB5791, PRJEB5299, PRJEB5320, PRJEB5312 and PRJEB5321.

## Author Contributions

J.G., C.P. and V.B. performed sequencing; M.L. and S.B. processed all NGS reads; M.L., S.B. and A.T. identified mutations; M.D. and S.K. generated samples; J.C. and U.G. conceived of the experiment; J.C. and Ö.T. wrote the manuscript. All authors approved the final manuscript.

## Supplementary Material

Supplementary Information Supplementary file 1

## References

[b1] Sjoblom T. *et al.* The consensus coding sequences of human breast and colorectal cancers. Science 314, 268–274 (2006). 16959974 10.1126/science.1133427

[b2] Greenman C. *et al.* Patterns of somatic mutation in human cancer genomes. Nature 446, 153–158 (2007). 17344846 10.1038/nature05610 PMC2712719

[b3] Allegra C. J. *et al.* American Society of Clinical Oncology provisional clinical opinion: testing for KRAS gene mutations in patients with metastatic colorectal carcinoma to predict response to anti-epidermal growth factor receptor monoclonal antibody therapy. J Clin Oncol 27, 2091–2096 (2009). 19188670 10.1200/JCO.2009.21.9170

[b4] Chang Y. F., Imam J. S. & Wilkinson M. F. The nonsense-mediated decay RNA surveillance pathway. Annual review of biochemistry 76, 51–74 (2007). 10.1146/annurev.biochem.76.050106.093909 17352659

[b5] Cartegni L., Chew S. L. & Krainer A. R. Listening to silence and understanding nonsense: exonic mutations that affect splicing. Nat Rev Genet 3, 285–298 (2002). 11967553 10.1038/nrg775

[b6] Shin N. *et al.* Identification of frequently mutated genes with relevance to nonsense mediated mRNA decay in the high microsatellite instability cancers. Int J Cancer 128, 2872–2880 (2011). 20824714 10.1002/ijc.25641

[b7] Adey A. *et al.* The haplotype-resolved genome and epigenome of the aneuploid HeLa cancer cell line. Nature 500, 207–211 (2013). 23925245 10.1038/nature12064 PMC3740412

[b8] Zhao X. *et al.* An integrated view of copy number and allelic alterations in the cancer genome using single nucleotide polymorphism arrays. Cancer Res 64, 3060–3071 (2004). 15126342 10.1158/0008-5472.can-03-3308

[b9] Koboldt D. C. *et al.* VarScan 2: somatic mutation and copy number alteration discovery in cancer by exome sequencing. Genome Res 22, 568–576 (2012). 22300766 10.1101/gr.129684.111 PMC3290792

[b10] Gandhi J. *et al.* Alterations in genes of the EGFR signaling pathway and their relationship to EGFR tyrosine kinase inhibitor sensitivity in lung cancer cell lines. PLoS One 4, e4576 (2009). 19238210 10.1371/journal.pone.0004576 PMC2642732

[b11] Ha G. *et al.* Integrative analysis of genome-wide loss of heterozygosity and monoallelic expression at nucleotide resolution reveals disrupted pathways in triple-negative breast cancer. Genome Res 22, 1995–2007 (2012). 22637570 10.1101/gr.137570.112 PMC3460194

[b12] Carter S. L. *et al.* Absolute quantification of somatic DNA alterations in human cancer. Nat Biotechnol 30, 413–421 (2012). 22544022 10.1038/nbt.2203 PMC4383288

[b13] Soh J. *et al.* Oncogene mutations, copy number gains and mutant allele specific imbalance (MASI) frequently occur together in tumor cells. PLoS One 4, e7464 (2009). 19826477 10.1371/journal.pone.0007464 PMC2757721

[b14] Crisan A. *et al.* Mutation discovery in regions of segmental cancer genome amplifications with CoNAn-SNV: a mixture model for next generation sequencing of tumors. PLoS One 7, e41551 (2012). 22916110 10.1371/journal.pone.0041551 PMC3420914

[b15] Nik-Zainal S. *et al.* The life history of 21 breast cancers. Cell 149, 994–1007 (2012). 22608083 10.1016/j.cell.2012.04.023 PMC3428864

[b16] Van Loo P. *et al.* Allele-specific copy number analysis of tumors. Proc Natl Acad Sci U S A 107, 16910–16915 (2010). 20837533 10.1073/pnas.1009843107 PMC2947907

[b17] Pastinen T. Genome-wide allele-specific analysis: insights into regulatory variation. Nat Rev Genet 11, 533–538 (2010). 20567245 10.1038/nrg2815

[b18] Babak T. *et al.* Global survey of genomic imprinting by transcriptome sequencing. Curr Biol 18, 1735–1741 (2008). 19026546 10.1016/j.cub.2008.09.044

[b19] Tuch B. B. *et al.* Tumor transcriptome sequencing reveals allelic expression imbalances associated with copy number alterations. PLoS One 5, e9317 (2010). 20174472 10.1371/journal.pone.0009317 PMC2824832

[b20] Li H. & Durbin R. Fast and accurate short read alignment with Burrows-Wheeler transform. Bioinformatics 25, 1754–1760 (2009). 19451168 10.1093/bioinformatics/btp324 PMC2705234

[b21] Dobin A. *et al.* STAR: ultrafast universal RNA-seq aligner. Bioinformatics 29, 15–21 (2013). 23104886 10.1093/bioinformatics/bts635 PMC3530905

[b22] Li H. *et al.* The Sequence Alignment/Map format and SAMtools. Bioinformatics 25, 2078–2079 (2009). 19505943 10.1093/bioinformatics/btp352 PMC2723002

[b23] Cibulskis K. *et al.* Sensitive detection of somatic point mutations in impure and heterogeneous cancer samples. Nat Biotechnol 31, 213–219 (2013). 23396013 10.1038/nbt.2514 PMC3833702

[b24] Larson D. E. *et al.* SomaticSniper: identification of somatic point mutations in whole genome sequencing data. Bioinformatics 28, 311–317 (2012). 22155872 10.1093/bioinformatics/btr665 PMC3268238

[b25] Castle J. C. *et al.* DNA copy number, including telomeres and mitochondria, assayed using next-generation sequencing. BMC Genomics 11, 244 (2010). 20398377 10.1186/1471-2164-11-244 PMC2867831

[b26] Alexandrov L. B. *et al.* Signatures of mutational processes in human cancer. Nature 500, 415–421 (2013). 23945592 10.1038/nature12477 PMC3776390

[b27] Castle J. C. *et al.* Immunomic, genomic and transcriptomic characterization of CT26 colorectal carcinoma. BMC Genomics 15, 190 (2014). 24621249 10.1186/1471-2164-15-190 PMC4007559

[b28] Duitama J., Srivastava P. & Mandoiu I. Towards accurate detection and genotyping of expressed variants from whole transcriptome sequencing data. BMC Genomics 13, (2012). 10.1186/1471-2164-13-S2-S6 PMC3394419 22537301

[b29] Stevenson K. R., Coolon J. D. & Wittkopp P. J. Sources of bias in measures of allele-specific expression derived from RNA-seq data aligned to a single reference genome. BMC Genomics 14, 536 (2013). 23919664 10.1186/1471-2164-14-536 PMC3751238

[b30] Li G. *et al.* Identification of allele-specific alternative mRNA processing via transcriptome sequencing. Nucleic Acids Res 40, e104 (2012). 22467206 10.1093/nar/gks280 PMC3401465

[b31] Glaser R. L., Ramsay J. P. & Morison I. M. The imprinted gene and parent-of-origin effect database now includes parental origin of de novo mutations. Nucleic Acids Res 34, D29–31 (2006). 16381868 10.1093/nar/gkj101 PMC1347463

[b32] Castle J. C. *et al.* Exploiting the mutanome for tumor vaccination. Cancer Res 72, 1081–1091 (2012). 22237626 10.1158/0008-5472.CAN-11-3722

